# Feasibility and Effectiveness of Indicator Condition-Guided Testing for HIV: Results from HIDES I (HIV Indicator Diseases across Europe Study)

**DOI:** 10.1371/journal.pone.0052845

**Published:** 2013-01-15

**Authors:** Ann K. Sullivan, Dorthe Raben, Joanne Reekie, Michael Rayment, Amanda Mocroft, Stefan Esser, Agathe Leon, Josip Begovac, Kees Brinkman, Robert Zangerle, Anna Grzeszczuk, Anna Vassilenko, Vesna Hadziosmanovic, Maksym Krasnov, Anders Sönnerborg, Nathan Clumeck, José Gatell, Brian Gazzard, Antonella d’Arminio Monforte, Jürgen Rockstroh, Jens D. Lundgren

**Affiliations:** 1 Directorate of Sexual Health and HIV Medicine, Chelsea and Westminster NHS Foundation Trust, London, United Kingdom; 2 Copenhagen HIV Programme, University of Copenhagen, Copenhagen, Denmark; 3 Department Infection of Population Health, University College London, London, United Kingdom; 4 Clinic of Dermatology and Venerology, University Hospital Essen, Essen, Germany; 5 Department of Infectious Diseases, Hospital Clínic de Barcelona, Barcelona, Spain; 6 University Hospital of Infectious Diseases, Zagreb, Croatia; 7 Department Internal Medicine, Onze Lieve Vrouwe Gasthuis, Amsterdam, The Netherlands; 8 Department of Dermatology and Venereology, Innsbruck Medical University, Innsbruck, Austria; 9 Department of Infectious Diseases and Hepatology, Medical University of Bialystok, Bialystok, Poland; 10 Department of Infectious Diseases, Belorussian State Medical University, Minsk, Belarus; 11 Clinical Center, Infectious Diseases Clinic, University of Sarajevo, Sarajevo, Bosnia and Herzegovina; 12 Kharkov Regional Clinic of Infectious Diseases, Kharkov, Ukraine; 13 Department of Infectious Diseases, Karolinska University Hospital, Stockholm, Sweden; 14 Department of Infectious Diseases, Saint-Pierre University Hospital, Brussels, Belgium; 15 Unit of Infectious Diseases, San Paolo Hospital, University of Milan, Milan, Italy; 16 Department of Medicine I, University of Bonn, Bonn, Germany; 17 Department of Infectious Diseases, Rigshospitalet, Copenhagen, Denmark; University College London Institute of Child Health, United Kingdom

## Abstract

Improved methods for targeting HIV testing among patients most likely to be infected are required; HIDES I aimed to define the methodology of a European wide study of HIV prevalence in individuals presenting with one of eight indicator conditions/diseases (ID); sexually transmitted infection, lymphoma, cervical or anal cancer/dysplasia, herpes zoster, hepatitis B/C, mononucleosis-like illness, unexplained leukocytopenia/thrombocytopenia and seborrheic dermatitis/exanthema, and to identify those with an HIV prevalence of >0.1%, a level determined to be cost effective. A staff questionnaire was performed. From October 2009– February 2011, individuals, not known to be HIV positive, presenting with one of the ID were offered an HIV test; additional information was collected on previous HIV testing behaviour and recent medical history. A total of 3588 individuals from 16 centres were included. Sixty-six tested positive for HIV, giving an HIV prevalence of 1.8% [95% CI: 1.42–2.34]; all eight ID exceeded 0.1% prevalence. Of those testing HIV positive, 83% were male, 58% identified as MSM and 9% were injecting drug users. Twenty percent reported previously having potentially HIV-related symptoms and 52% had previously tested HIV negative (median time since last test: 1.58 years); which together with the median CD4 count at diagnosis (400 cell/uL) adds weight to this strategy being effective in diagnosing HIV at an earlier stage. A positive test was more likely for non-white individuals, MSM, injecting drug users and those testing in non-Northern regions. HIDES I describes an effective strategy to detect undiagnosed HIV infection. All eight ID fulfilled the >0.1% criterion for cost effectiveness. All individuals presenting to any health care setting with one of these ID should be strongly recommended an HIV test. A strategy is being developed in collaboration with ECDC and WHO Europe to guide the implementation of this novel public health initiative across Europe.

## Background

Of the estimated 2.3 million HIV infected individuals living in the European Region, it is estimated that one in three are unaware of their diagnosis [1]–[3]. Among those diagnosed with HIV, 50% have a CD4 count <350 cells/uL at diagnosis, negatively impacting on both individual and public health [4], [5]. Late diagnosis is associated with increased morbidity and mortality [6]–[9], poorer response to antiretroviral treatment [10]–[12], increased rates of HIV transmission [13] and increased healthcare costs [14], [15]. A critical public health issue is, therefore, how to diagnose individuals with HIV infection at an earlier stage of disease. This will require the introduction of innovative approaches to better target testing for those most likely to be infected with HIV and who present late for care.

Certain “indicator” conditions occur more frequently in HIV-infected individuals, either because they share a common mode of transmission or because their occurrence is facilitated by the characteristic immune deficiency associated with HIV infection. There is little evidence, however, regarding the prevalence of HIV infection in individuals of unknown HIV status presenting for care with such conditions [16]. Data from the US and France [17], [18], [19] suggest that delivering testing in settings with an HIV prevalence of at least 0.1% is cost effective. Some testing guidelines now promote indicator condition guided HIV testing as part of an overall provider initiated testing strategy [20], [21]. However, little evidence exists to support such a strategy. In response to the sustained high levels of undiagnosed HIV across Europe, consensus was reached at the HIV in Europe 2007 Conference [16], to propose an indicator condition guided testing approach. The HIDES study (HIV Indicator Diseases across Europe Study) was subsequently designed by the HIV in Europe initiative (www.hiveurope.eu).

The overall objective of HIDES was to identify those indicator diseases/conditions (ID) with an HIV prevalence of >0.1% and to ascertain whether there is variation in prevalence across Europe. HIDES I aimed to define the methodology of such an approach, to determine the HIV prevalence of eight pre-selected ID, and to describe the characteristics of those testing HIV positive, their previous HIV testing behaviour and whether missed opportunities for earlier diagnosis could be identified.

## Methods

Eight indicator conditions were selected for inclusion based on a combination of factors including: a significant health risk if HIV remained undiagnosed, the opportunity of identifying early infection/seroconversion, the likelihood of a high HIV prevalence in the ID, or expert opinion underpinned by clinical experience and best available evidence [16], [20], [21]. TB and other AIDS defining illnesses were not included as there is already wide spread acceptance of the need for HIV testing in these conditions. Given the paucity of data regarding HIV prevalence for other conditions, the focus of this study was on other IDs to provide the evidence of HIV prevalence >0.1%.

Recruitment was by a call for expression of interest to more than 200 healthcare centres across Europe; healthcare settings were eligible for consideration if they handled one or more of the following eight conditions as part of their routine delivery of care:

- Sexually transmitted infections (STI)
- Malignant lymphoma, irrespective of type (LYM)
- Cervical or anal cancer/dysplasia (CAN)
- Herpes zoster (HZV)
- Hepatitis B or C virus infection, acute or chronic, and irrespective of time of diagnosis relative to survey (HEP)
- Ongoing mononucleosis-like illness (MON)
- Unexplained leukocytopenia/thrombocytopenia lasting >4 weeks (CYT)
- Seborrheic dermatitis/exanthema (SEB)

Thirty-five centres responded to the call for collaboration. Seventeen centres were excluded due to very low expected recruitment numbers and two were subsequently unable to start prospective recruitment due to administrative obstacles. Centres were selected based on the estimated number of presenting cases of the relevant ID and the predicted recruitment time; selection was also based on the aim to deliver a balanced number of surveys per ID within the study overall. Therefore each centre was invited to recruit only to a proportion of the eight IDs. One survey was defined as prospectively delivered routine HIV testing for a single ID at each individual centre. Ethical approval was obtained in all participating countries in line with their National standards. In the majority of settings, where relevant, training for delivering HIV testing was delivered to local staff, and resources made available for support and referral. In two settings specific training was not delivered, but support was provided or the STI/HIV clinic staff did the testing.

Consecutive patients were enrolled from October 2009– February 2011 if they presented with the selected ID and were not already known to be HIV positive. Patient inclusion was based on the treating physician’s clinical, microbiological or histological diagnosis. All patients were offered an HIV test and the outcome of the test was recorded. Centres could use either rapid point of care or serological HIV tests. Additional information, including demographics, previous HIV testing behaviour and relevant past medical history (previous AIDS-related symptoms), hospital admissions, and prior or past STI and viral hepatitis testing behaviour was collected by the treating health care provider. Data were transmitted to the central co-ordinating centre for entry and analysis.

In addition, a questionnaire was completed by survey coordinators and clinical staff delivering the testing, at its conclusion. This examined the attitudes of health care workers towards routine HIV testing for the specific ID and explored the challenges encountered. Unpublished prevalence data was collected from participating study sites for comparison with the prevalence found in the IDs.

For the purposes of this analysis patients were divided into four geographic regions as previously defined by EuroSIDA [22]: North (Denmark, Sweden, Netherlands, UK), West Central (Austria, Belgium, Germany), East Central (Belarus, Bosnia, Croatia, Poland, Ukraine) and South (Italy and Spain).

Patient characteristics were compared using the Chi-squared test for categorical, and the Kruskall-Wallis test for continuous, variables. The exact binomial method was used to calculate the 95% confidence intervals for HIV prevalence for each ID. Logistic regression was used to investigate which factors were associated with a HIV-positive diagnosis in this study. Each factor was fitted individually into a univariable model. Multivariable models were developed using variables found to be significant (p<0.10) in univariable analyses. A stepwise selection method was used to confirm final model selection. All analyses were performed using SAS version 9.2 [23].

## Results

### Centre Characteristics

Thirty-nine surveys were thus conducted at 16 sites in 14 countries. All centres were urban, hospital based services, except for Barcelona, where recruitment took place at four primary care units collaborating with the hospital, and Brussels, where recruitment was in both hospital and primary care.

Twelve surveys were carried out in outpatient departments, three in inpatient settings, eight in a combination of outpatient and inpatient, four in primary care and two in a combination of outpatient clinic and primary care. Before the HIDES study was introduced, testing for HIV was considered routine in less than half the settings (11/29). Local clinic staff performed the HIV test in 23 of the surveys, in one survey STI/HIV clinic staff did the testing, and in eight surveys it was a combination of local clinical staff and STI/HIV staff.

### Patient Characteristics

A total of 3588 individuals accepted the offer of an HIV test. Patient characteristics are detailed in Table 1, according to the region of enrolment. The highest proportion of individuals was recruited in East Central Europe (n = 1412, 39%), followed by North Europe (n = 1288, 36%). In North Europe, the median age was lower compared to the other regions, and a higher proportion of females were recruited. In East Central almost all the individuals enrolled reported their sexual orientation as heterosexual (97%) compared to approximately 60% in other regions. A lower proportion of individuals in East Central Europe (13%) reported having had a previous HIV test.

**Table 1:** Patient characteristics by European region.

		European Region^1^				
		North	West Central	East Central	South	p-value
Total (N,%)		1288 (35.9)	459 (12.8)	1412 (39.4)	429 (12.0)	
Gender (N,%)	Male	567 (44.0)	302 (65.8)	817 (57.9)	292 (68.1)	<.0001
	Female	716 (55.6)	157 (34.2)	591 (41.9)	136 (31.7)	
Ethnicity	White	999 (77.6)	288 (84.7)	1390 (99.0)	385 (89.7)	<.0001
Age (median IQR^2^)		33 (28–46)	37 (28–47)	37 (26–51)	43 (32–59)	<.0001
Sexual orientation (N,%)	Heterosexual	717 (55.7)	274 (59.7)	1365 (96.7)	263 (61.3)	<.0001
	Homosexual/bisexual	146 (11.3)	32 (7.0)	19 (1.3)	93 (21.7)	
	Unknown	425 (33.0)	153 (33.3)	28 (1.9)	73 (17.0)	
Previous HIV test (N,%)		743 (57.7)	209 (45.5)	185 (13.1)	153 (35.6)	<.0001
Indicator condition^3^(N,%)	STI	526 (40.8)	103 (22.4)	0 (0.0)	135 (31.5)	<.0001
	LYM	88 (6.8)	6 (1.3)	250 (17.7)	0 (0.0)	
	CAN	374 (29.0)	122 (26.6)	46 (3.3)	0 (0.0)	
	HZV	0 (0.0)	102 (22.2)	84 (6.0)	21 (4.9)	
	HEP	291 (22.6)	68 (14.8)	606 (42.9)	134 (31.2)	
	MON	0 (0.0)	2 (0.4)	414 (29.3)	25 (5.8)	
	CYT	4 (0.3)	0 (0.0)	12 (0.9)	78 (18.2)	
	SEB	5 (0.4)	56 (12.2)	0 (0.0)	36 (8.4)	
Number tested HIV positive (N,%)		8 (0.6)	7 (1.5)	23 (1.6)	28 (6.5)	<.0001

1 North:Denmark, Sweden, Netherlands, UK, West Central: Austria, Belgium, Germany, East Central: Belarus, Bosnia, Croatia, Poland, Ukraine and South: Italy and Spain.

2 IQR:Interquartile range.

3 STI: Sexually transmitted infection, LYM: Malignant lymphoma, CAN: Cervical or anal cancer/dysplasia, HZV: Herpes zoster, HEP: Hepatitis B or C, MON: Ongoing mononucleosis-like illness, CYT: Unexplained leukocytopenia/thrombocytopenia lasting >4 weeks, SEB: Seborrheic dermatitis/exanthema (SEB).

Missing data: 10 (0.3%) gender, 120 (3.3%) ethnicity, 63 (1.8%) age, 120 (3.3%) sexual orientation, 291 (8.1%) previous HIV test.

### HIV Prevalence by Indicator Condition

Of the 3588 individuals included, sixty-six were diagnosed with HIV, yielding an overall prevalence of 1.8% [95% CI: 1.42–2.34]. Table 2 indicates the prevalence of HIV by ID. All eight ID individually fulfilled the study’s criteria of demonstrating an HIV prevalence of >0.1%, however, for LYM and CAN, 0.1 fell within the 95% confidence interval. The highest prevalence was found in STI: 4.06% [95% CI 2.78–5.71].

**Table 2:** Prevalence of HIV by indicator condition.

	Individuals having HIV test(number)	HIV positive(number)	Prevalence(95% CI )	Number of surveys	Local HIV prevalence*	Country HIV prevalence***
Total	3588	66	1.84 (1.42–2.34)	39		0.1–1.1
Indicator condition						
Sexually transmitted infection (STI)	764	31	4.06 (2.78–5.71)	4	0.8–3.0	0.2–0.3
Malignant lymphoma (LYM)	344	1	0.29 (0.006–1.61)	5	0.8	0.1–0.2
Cervical or anal dysplasia or cancer (CAN)	542	2	0.37 (0.04–1.32)	4	0.8	0.1–0.2
Herpes zoster (HZV)	207	6	2.89 (1.07–6.21)	5	0.3–0.9	0.1–0.4
Hepatitis B or C (HEP)	1099	4	0.36 (0.10–0.93)	6	0.2–2.8**	0.1–1.1
Ongoing mononucleosis-like illness (MON)	441	17	3.85 (2.26–6.10)	7	0.2–0.9	0.3–1.1
Unexplained leukocytopenia/thrombocytopenia (CYT)	94	3	3.19 (0.66–9.04)	4	0.3–0.8	0.1–0.4
Seborrheic dermatitis/exanthema (SEB)	97	2	2.06 (0.25–7.24)	4	0.3–0.8	0.2–0.4

* Unpublished prevalence data from participating study sites.

** includes MSM, IDU prevalence.

*** UNAIDS adults aged 15–49 country HIV prevalence rate [31].

Table 3 shows the HIV prevalence according to ethnicity (stratified as white *vs.* non-white) and by region of recruitment. The prevalence of HIV was highest in Southern Europe, where an HIV prevalence of >5% was observed in both whites and non-whites, and lowest in North Europe (<1%). Of the 2348 individuals who identified as heterosexual, white and non-IDU (Intravenous Drug User) (*i.e*. demographically ‘lower risk’), 19 tested positive, giving an HIV prevalence of 0.81% (95% CI 0.48–1.26).

**Table 3:** Prevalence of HIV-positive test by ethnicity and region.

	North	West Central	East Central	South
Total number of tests (%)	1288 (35.9)	459 (12.8)	1412 (39.4)	429 (12.0)
Number tested HIV positive	8 (0.6)	17 (1.5)	23 (1.6)	28 (6.5)
White	0.60 (0.22–1.30)	1.04 (0.21–3.01)	1.65 (1.06–2.47)	6.23 (4.03–9.14)
non-white	0.69 (0.08–2.47)	2.34 (0.64–5.88)	0 (0–15.4)	9.09 (2.53–21.7)

### Characteristics of Individuals who Tested Positive for HIV, and Potential Missed Opportunities for Earlier Diagnosis

Of patients testing HIV positive 83.3% were male, 85.0% were white, 57.6% identified themselves as homo- or bi-sexual and 9.1% as injecting drug users. More than half (51.5%) had previously tested HIV negative; the median time since the last HIV test was 1.6 years (range 0.05–12.71 years). In the preceding five years, 27 (54.0%) individuals had made at least one visit to a sexual health clinic, 13 (19.7%) had experienced potential HIV-related symptoms, and 7 (10.0%) individuals had been admitted to hospital, the majority with a potential AIDS diagnosis or infection. The presenting CD4 T-cell count was available for 35 (53%) individuals who tested HIV-positive, with a median of 400 cells/µL (range 11–675).

### Predictors of Testing HIV Positive

In unadjusted analysis, individuals presenting with LYM (OR 0.07, 95%CI 0.009–0.51, p = 0.008), CAN (OR, 0.09, 95%CI 0.02–0.37, p = 0.0009), and HEP (0.09, 95%CI 0.03–0.25), p<.0001) were less likely to test HIV positive than those presenting with STI. There was no significant difference in the odds of testing HIV positive in those presenting with HZV (p = 0.44), MON (p = 0.86), CYT (p = 0.69) or SEB (p = 0.34) and those with STI. After adjustment for ethnicity, sexual orientation, active IDU status, whether or not the individual had previously had tested for hepatitis B, and region of Europe, there were no significant differences between presenting ID in odds of testing HIV positive.

**Figure 1:**
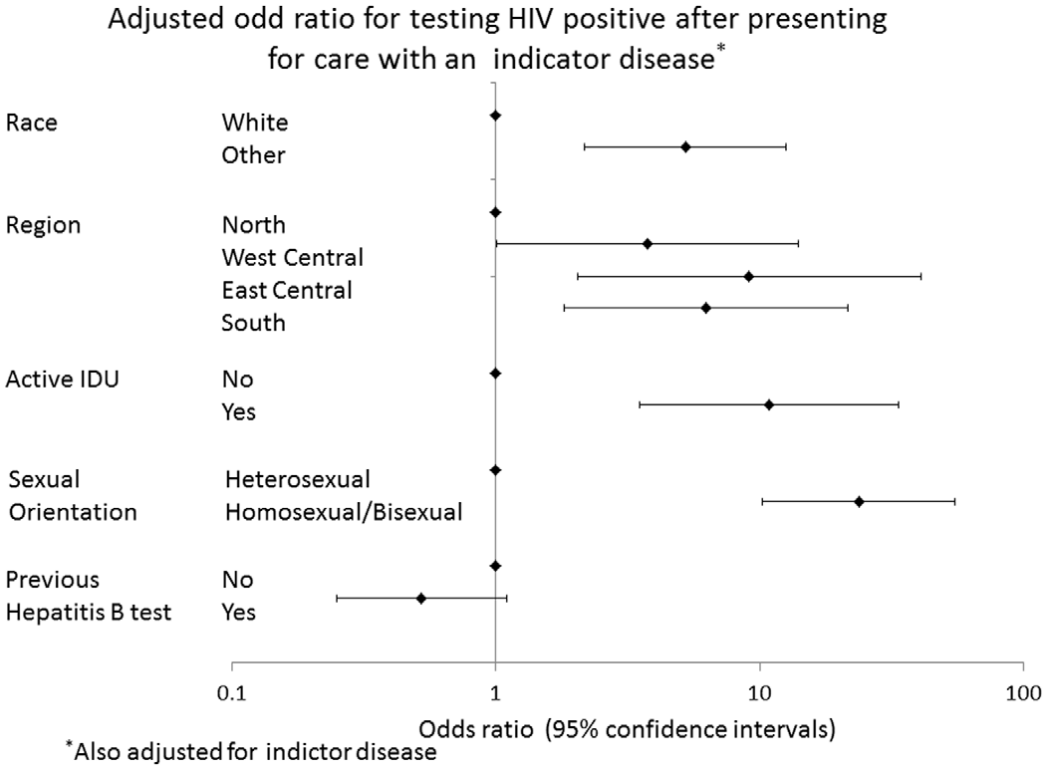
Odd ratio for testing HIV positive.

Factors that were independently associated with testing HIV positive are shown in Figure 1. Individuals who were of non-white ethnic origin (adjusted odds ratio [OR] 4.73, 95%CI 2.16–12.61,p = 0.0002), homosexual/bisexual (aOR 23.72, 95%CI 10.20–55.17, p<.0001) or active IDU (aOR 10.86, 95%CI 3.52–33.50, p<0.0001) were more likely to test HIV positive. Additionally, individuals enrolled from West Central (aOR 3.76, 95% CI 1.01–14.04, p = 0.04), East Central (aOR 9.12 95%CI 2.04–40.79, p = 0.003) or South (aOR 6.25, 95% CI 1.81–21.52, p = 0.003) were more likely to test HIV positive compared to individuals from North Europe.

### Acceptability of HIV Test Offer

For those surveys where the proportion of eligible patients offered an HIV test is available (7 centres, 16 surveys) median coverage in hospital settings was 92% (range 66–100%), and 12% in primary care (Chi square p-value <0.0001). The median uptake in both settings was 96% (range 62–100%). Due to difficulty with data collection/disease reporting at some centres, this data is not available for all surveys.

### Questionnaire Study

Thirty of 39 questionnaires were received from survey coordinators (77% return rate). Fifty-three percent reported they perceived that motivation of colleagues was a barrier to the implementation of the surveys. When asked about colleagues’ attitudes towards the study, 10% said they thought their colleagues were positive, 43% considered they were neutral and 36% felt they were sceptical.

Sixty-two short questionnaires were received from the clinical staff delivering the testing. Time pressure was reported by 47% as a barrier to offering an HIV test as part of routine care, 13% had concerns they would be asked difficult questions, 10% felt deskilled and 7% expressed a need for additional training. Reasons for not offering a test routinely were reported to be due to lack of time or because the patient was not perceived to be at risk (both at 32%), because they forgot (25%), or because the patient reported a recent test (16%).

## Discussion

For the first time, HIDES I has demonstrated an effective indicator condition guided HIV testing strategy in Europe, effectively diagnosing individuals with HIV infection. Phase I of the study has demonstrated proof of concept, showing that such an approach is both feasible and acceptable.

All eight IDs individually fulfilled the criteria of demonstrating an HIV prevalence of >0.1%, with some variation. Despite the recognition that these IDs are related to HIV infection and occur more commonly in those infected, there are little data on previously undiagnosed HIV prevalence among individuals presenting with these conditions. Implementing this strategy across Europe would have significant resource implications. However, data from the US and France propose testing to be cost effective if it is delivered at an HIV positivity rate of 0.1% [17], [18], [19], and the results of HIDES I indicate this cut off is likely to be achieved for each of the eight clinical conditions investigated. However, there may be a degree of variation across Europe in regard to health care utilization and costs, and further country specific data are required to analyse cost effectiveness. Furthermore, in two of the conditions, the target fell within the 95% CI. Increased recruitment through the planned second phase (HIDES II) should be able to further address these issues.

The high HIV prevalence in individuals presenting with a STI is expected, as they share their route of transmission, and the prevalence approaches those rates reported elsewhere (8.8–15.1% among MSM attending UK GUM clinics, for example) [24]. However the high detection rate in STI in HIDES I, is also likely to be influenced by the fact that clinics participating in the STI surveys routinely offer HIV tests to all attending individuals regardless of presenting complaint. This is likely to increase both the offer rate and the uptake of testing, as patients expect to be offered a test in this setting, and tests are offered by specialist clinicians in a routine manner.

The HIV prevalence in individuals presenting with a mononucleosis-like illness was also high at 3.85%, and it is possible these symptoms represented HIV sero-conversion. Diagnosis at sero-conversion has the added advantage of enabling an individual to be aware of their HIV status at their most infectious stage, and thus timely diagnosis may potentially impact on levels of onward transmission [25].

Furthermore, a significant number of patients diagnosed with HIV in the study overall had previously tested HIV negative, and the short median time since their last negative test would suggest early diagnosis had occurred in many of these individuals by utilising this strategy. Additionally, the presenting median CD4 count for all patients diagnosed with HIV in the study is higher than that reported at national levels [1], [26] and adds further weight to this strategy being effective in diagnosing HIV infection at an earlier stage.

The differences in the patient characteristics observed are likely to be due, in part, to different surveys being carried out in different regions: 40% of individuals in Northern Europe were included in a STI survey compared to none in East Central Europe; the majority (43%) of individuals recruited in East Central Europe were included in a hepatitis survey. Of individuals in East Central Europe, 29% were included in a MON survey, significantly higher compared to the other regions. This is due to the methodology, whereby each centre only recruited to a proportion of the eight ID (range 1–5), and the IDs were self-selected.

Although the odd ratio of testing HIV positive was higher in those with well-recognised risk-factors (MSM, non-white ethnicity and IDU) individuals presenting with an ID but not from these groups had an HIV prevalence of 0.81%, still far exceeding the 0.1% target. Given that risk-based targeted testing as a strategy to date has not been sufficiently effective, and that in many health care settings information about risk factors is not sought or elicited, indicator condition guided HIV testing is a worthy additional strategy to enhance current testing programmes.

The uptake rates reported suggest the strategy is acceptable to patients regardless of setting or ID, and is higher than that reported elsewhere [27], [28]. The offer rate, where available, varies significantly between acute and primary care settings. The offer rate in acute settings is higher than that reported elsewhere [27], [28]; in primary care it is lower than some reports [27], [28], however it is similar to some experience outside research studies [unpublished data]. It may be the offer rate is a surrogate marker of the strategy’s acceptability to staff, or it may represent organisational factors within primary care impacting on feasibility. This discrepancy warrants further study.

Staff involved in the study identified major barriers to delivering ID guided testing, including operational, attitudinal barriers, and training needs: however in the majority of settings this was not reflected in light of the high offer and uptake rates. Some of this may be due to study staff incorrectly attributing attitudes to local clinic staff; however the clinic staff themselves did report a high level of predominantly operational barriers, along with perceptions of low risk. This is similar to that reported elsewhere [27], [29] and poses a significant challenge to rolling out any strategy calling for increased testing and aiming to ‘normalise’ the offer of an HIV test. It is possible that the introduction of indicator condition guided testing could remove some of the health provider barriers to the offer of a test. Removing the need for risk assessment and making indicator conditions a trigger for the routine offer of an HIV test has the potential to reduce HIV related stigma and discrimination and increase the number of tests offered and accepted, thus ‘normalising’ HIV testing.

Table 2 shows prevalence data (where available) for the locations in which the surveys were conducted. In the majority of cases (6/8) the prevalence identified through an ID driven strategy exceeds the local prevalence, suggesting the strategy would be an effective and efficient way of identifying HIV infection, thereby helping to reduce the burden of undiagnosed disease. In one ID, hepatitis, the prevalence range includes prevalence figures for IDU and MSM populations - both high prevalence populations themselves. In the remaining two ID, lymphoma and CIN, the local prevalence data is only available for one site, which contributed less than half the HIV tests, which therefore makes comparison less reliable.

The comparator data are derived mainly from national surveillance data. Other investigational testing strategies reveal differing results, depending on whether they are targeted or general screening HIV programmes in high prevalence areas. These latter, general programmes largely produce lower prevalence figures than our ID driven testing. [29], [30].

It is likely that the findings of this study overall are generalizable across the European region, albeit requiring some local adaptation. Acceptability, for example, is highly likely to be adversely influenced by issues such as stigma, and access to treatment and care. Prevalence in hepatitis will be affected by levels of IDU and access to prevention programmes such as needle exchange. The healthcare setting may also influence final prevalence rates with different severity and/or chronicity of symptoms impacting on the likelihood of underlying HIV infection. However data to date would suggest, even allowing for such regional differences, the resultant prevalence is likely still to remain above 0.1%. Close monitoring and early review after introducing such a strategy would inform the value of its continuation.

Clinics are encouraged to report audits on HIV prevalence for the various IDs and to implement surveys for new ID conditions through the HIDES II study and the HIV in Europe initiative (www.hiveurope.eu).

### Limitations

The relatively small number of patients enrolled in HIDES I has resulted in wide CI around the prevalence estimates for some ID. However the main aim of HIDES I was to define the methodology and ascertain the acceptability and feasibility of the strategy of indicator condition guided HIV testing; with further recruitment to HIDES II more precise prevalence data should appear. There was limited information for individuals testing HIV positive regarding CD4 count at diagnosis, disease stage and access to care; this will be an additional outcome in the second phase of the study. Finally, there was very limited data available on those who declined HIV testing or were not offered a test, which may have introduced bias, as those who declined may have done so already knowing their status. Additionally, staff may have introduced bias by targeting testing on the basis of perceived risk, either high or low.

### Conclusions

Indicator condition guided HIV testing is an acceptable, feasible and effective strategy to address the on-going HIV epidemic in Europe by reducing the level of undiagnosed HIV infection, and potentially facilitates earlier diagnosis. In line with ECDC (European Centre for Disease Prevention and Control) guidance, this study demonstrates that individuals presenting to any healthcare setting with any of the eight indicator conditions should be strongly recommended to have an HIV test.

The study has also reiterated that potential barriers to routine testing still exist with clinicians, and this requires urgent address through collaboration and information. A strategy is currently being developed in collaboration with ECDC and WHO Europe aimed at guiding the implementation of this novel public health initiative across Europe. The follow up study, HIDES II, will expand this testing strategy by increasing the number of indicator conditions and centres involved.
